# miR-143-3p impacts on pulmonary inflammatory factors and cell apoptosis in mice with mycoplasmal pneumonia by regulating TLR4/MyD88/NF-κB pathway

**DOI:** 10.1042/BSR20193419

**Published:** 2020-07-07

**Authors:** Yongjun Wang, Huan Li, Yongsheng Shi, Shuying Wang, Yan Xu, Hanyi Li, Donghai Liu

**Affiliations:** 1 Department of Pediatric Respiratory Medicine, Gansu Provincial Maternity and Child-Care Hospital, Lanzhou, Gansu Province, China; 2 Department of Rehabilitation, Gansu Province Hospital Rehabilitation Center, Lanzhou, Gansu Province, China

**Keywords:** apoptosis, inflammation, miR-143-3p, mycoplasmal pneumonia, TLR4/MyD88/NF-κB signaling pathway

## Abstract

miR-143-3p is correlated with inflammatory pain responses, such as hsa-miR-143-3p expression reduction in fibromyalgia. The present study aimed to explore the effects of miR-143-3p and Toll-like receptor (TLR) 4/myeloid differentiation factor 88 (MyD88)/NF-κB signaling pathway on pulmonary inflammatory factors levels and alveolar epithelial cell apoptosis in mycoplasmal pneumonia mice. Twenty mice were selected as normal group. The 120 successfully modeled *Mycoplasma pneumoniae* (MP) infection mice were randomly divided into model group (without any treatment), negative control (NC) group (injected with NC mimic), miR-143-3p mimic group (injected with miR-143-3p mimic), miR-143-3p inhibitor group (injected with miR-143-3p inhibitor), TAK-242 group (treatment with TAK-242), and miR-143-3p inhibitor + TAK-242 group (treatment with miR-143-3p inhibitor + TAK-242). Compared with model group, model mice had up-regulated miR-143-3p expression and decreased MyD88 and p-NF-κB p50 protein expressions (all *P*<0.05); Model mice treated with miR-143-3p mimic and TAK-242 had reduced interleukin (IL)-2 and tumor necrosis factor (TNF)-α contents and protein expressions of MyD88, p-NF-κB p50, increased IL-10 content, fewer alveolar epithelial cell apoptosis, lower Bax expression and higher Bcl-2 expression (all *P*<0.05); however, mice with miR-143-3p inhibitor treatment showed opposite trends in terms of above indicators. The exacerbation of mycoplasmal pneumonia caused by miR-143-3p inhibitor was partly improved by miR-143-3p inhibitor + TAK-242 combination treatment (all *P*<0.05). Therefore, up-regulation of miR-143-3p expression may ameliorate pulmonary inflammatory factors levels and reduce alveolar epithelial cell apoptosis in mycoplasmal pneumonia mice by inhibiting TLR4/MyD88/NF-κB signaling pathway.

## Introduction

*Mycoplasma pneumoniae* (MP) is a Gram-negative microorganism and the main cause of respiratory tract infection and community-acquired pneumonia [1], which is often associated with hemolysis, skin injury, arthralgia, gastrointestinal symptoms, central nervous system problems, heart disease and other extra-pulmonary complications [2,3]. MP is a prokaryotic human pathogen in the class Mollicutes and has key microbiological characteristics that differ from other bacteria. MP is the smallest self-replicating bacterium containing extremely small genome [4,5]. As a prokaryotic pathogen, it has three membranes and the absence of cell wall, and its survival is dependent on nutrition exchange of the host. MP has slow growth, of which the cultivation takes up to 6 weeks [6]. MP initially attaches to the surface of airway epithelial cells. The absence of cell wall facilitates MP membrane to directly contact its host, thus being able to transfer or exchange membrane components [7]. In the process, pathogen with toxic molecules destroys host cells and induces ciliary dyskinesia and epithalaxia to acquire key nutrients for growth [8]. Pathogenicity of *Mycoplasma* infection is the result of local tissue destruction, cytotoxicity and host immune response, and it spreads from person to person through respiratory droplets. Once MP attaches to epithelial cells, it will produce reactive oxygen species to damage epithelial cells [9].

MP triggers the production of interleukin (IL)-8, tumor necrosis factor (TNF)-α and other pro-inflammatory cytokines. Content of IL-8 and TNF-α in the serum increase with the aggravation of MP infection [10]. Several MP membrane proteins have a high affinity to receptors on host cells. *Mycoplasma* membrane lipoprotein induces host immune responses through interacting with pattern recognition receptors, especially Toll-like receptor (TLR) 2 and TLR6 [11]. TLR4/myeloid differentiation factor 88 (MyD88)/NF-κB signaling pathway participates in the body’s immune responses and alveolar inflammation. TLR4 is activated after body injury and further promotes the expression of downstream factors MyD88 and NF-κB, facilitating the expression of inflammatory factors IL-2 and TNF-α [12,13]. Alveolar epithelial cells synthesize and secrete pulmonary inflammation-related cytokines, and the apoptosis of alveolar epithelial cells leads to the development of pneumonia. Apoptosis is an important mechanism for losing defense function of body, which includes two main synergistic approaches, the external death receptor pathway and internal apoptosis signal pathway [14]. Cytokines play a vital role in the pathogenesis of pneumonia, which affects the intercellular signal transduction and inflammation. TNF, IL-1, IL-6, 1L-8, IL-10 and other anti-inflammatory cytokines are crucial to regulate immune responses [15].

As a non-coding RNA, microRNA (miRNA) can regulate and control multiple life activities by regulating the transcription of downstream target gene. We speculated that there might be some miRNAs that could regulate inflammatory cytokine release and alveolar epithelial cell apoptosis in mycoplasmal pneumonia by affecting TLR4/MyD88/NF-κB signaling pathway [16,17]. In the bioinformatics screening, we found that there was a targeted binding site of miR-143-3p and MyD88. miR-143-3p can inhibit the activation of extracellular signal-regulated protein kinase 5 (ERK5) and further damage the anti-inflammatory activity of PPARδ [18]. However, some studies report that miR-143-3p is down-regulated in cardiovascular diseases [19]. It is demonstrated that miR-143-3p is correlated with inflammatory pain responses, such as hsa-miR-143-3p expression reduction in fibromyalgia patients [15]. Therefore, we speculated that miR-143-3p might regulate MyD88/NF-κB signaling pathway to inhibit inflammatory factors’ levels and alveolar epithelial cell apoptosis in mice with mycoplasmal pneumonia by the targeted down-regulation of MyD88 expression.

Therefore, our study was aimed at exploring whether miR-143-3p could affect inflammatory factors’ levels and alveolar epithelial cell apoptosis in mice with mycoplasmal pneumonia by regulating TLR4/MyD88/NF-κB signaling pathway.

## Methods

### Laboratory animals

A total of 160 healthy male C57BL/6 mice (clean grade, weighing 35 ± 5 g) were regularly fed, of which 20 mice were in normal group, and the rest were used to establish the mycoplasmal pneumonia model. MP standard strain was re-dissolved in PPLO complete culture solution and incubated at 37°C in a biochemical incubator. MP infection model was established by nasal drip for 14 days. Mice in the normal group were administrated the same amount of distilled water. The present study was carried out in the Gansu Provincial Respiratory Endoscopy Medical Quality Control Center and approved by the Ethics Committee of Gansu Provincial Respiratory Endoscopy Medical Quality Control Center (9622018J0231).

### Grouping and treatment

The successfully modeled mice were divided into model group (without any treatment), negative control (NC) group (injection of NC mimic), miR-143-3p mimic group (injected with miR-143-3p mimic), miR-143-3p inhibitor group (injected with miR-143-3p inhibitor), TAK-242 group (injected with TLR4 inhibitor, TAK-242), and miR-143-3p inhibitor + TAK-242 group (injected with miR-143-3p inhibitor and TAK-242). NC, miR-143-3p mimic and miR-143-3p inhibitor were designed and synthesized by the Suzhou GenePharma Co., Ltd., China. TAK-242 (MedChemExpress Limited Liability Company) was diluted to 10 mg/ml and was intraperitoneally injected into mice at the dose of 10 mg/kg per 3 days for 2 weeks. Then mice were killed by collecting blood from the eyeball under narcotism by intraperitoneal injection of 0.3% pentobarbital sodium (30 mg/ kg), and the lung tissue was harvested and stored in liquid nitrogen.

### Dual-luciferase reporter system

The binding site between miR-143-3p and MyD88 was predicted by bioinformatics website (www.targetscan.org), which was verified by dual-luciferase reporter system assay. PGL3-MyD88 wildtype (wt), PGL3-MyD88 mutant (mut) reporter plasmids were constructed. The two reporter plasmids and *Renilla* luc plasmid were co-transfected with NC mimic and miR-143-3p mimic into HEK293T cells, respectively. Twenty-four hours after cell transfection, the *Renilla* luciferase activity were detected according to the instruction of dual-luciferase reporter kit (D0010, Solarbio, Beijing, China).

### qRT-PCR

RNA in the lung tissue of four mice in each group was extracted with TRIzol (Thermo Fisher Scientific, New York, U.S.A.). RNA was reverse-transcribed into cDNA by reverse transcription kit (Thermo Scientific, U.S.A.). qRT-PCR detection was performed by using SYBR® Premix Ex Taq™ II kit (TaKaRa, Dalian, China). qRT-PCR solutions included 2 μl PCR forward primer, 2 μl PCR reverse primer, 25 μl SYBR® Premix Ex Taq™ II (2×), 1 μl ROX Reference Dye (50×), 4 μl DNA templates and 16 μl ddH_2_O (Fortune Bio-tech Co., Ltd., Shanghai, China). Primers were synthesized by the BioSune Biotech Co., Ltd., Shanghai, China (Table 1). qRT-PCR was performed using ABI PRISM®7300 system (Prism®7300, Shanghai Kunke Equipment Co., Ltd., China). qRT-PCR conditions: pre-denaturation at 95°C for 10 min followed by 35 circles of denaturation at 95°C for 15 s and annealing at 60°C for 30 s, and extension at 72°C for 1 min. miR-143-3p took U6 as the internal reference, and others took GAPDH as the internal reference. 2^−ΔΔ*C*_t_^ showed the relative expression level of each target gene.

**Table 1:** qRT-PCR primer sequences

Gene	Sequence
*miR-143-3p*	Forward: 5′-TGAGATGAAGCACTGTAGCTC-3′
	Reverse: 5′-GCTACAGTGCTTCATCTCATT-3′
*TLR4*	Forward: 5′-ACAAACGCCGGAACTTTTCG-3′
	Reverse: 5′-GTCGGACACACACAACTTAAG-3′
*MyD88*	Forward: 5′-TTGCCAGCGAGCTAATTGAG-3′
	Reverse: 5′-ACAGGCTGAGTGCAAACTTG-3′
*NF-κB p50*	Forward: 5′-TGTCTGCACCTGTTCCAAAG-3′
	Reverse: 5′-TCAGCATCAAACTGCAGGTG-3′
*Bcl-2*	Forward: 5′-AGTACCTGAACCGGCATCTG-3′
	Reverse: 5′-GCTGAGCAGGGTCTTCAGAG-3′
*Bax*	Forward: 5′-CGAGCTGATCAGAACCATCA-3′
	Reverse: 5′-GGTCCCGAAGTAGGAGAGGA-3′
*U6*	Forward: 5′-CTCGCTTCGGCAGCACA-3′
	Reverse: 5′-AACGCTTCACGAATTTGCGT-3′
*GAPDH*	Forward: 5′-TCTCCCTCACAATTTCCATCCC-3′
	Reverse: 5′-TTTTTGTGGGTGCAGCGAAC-3′

### Western blot

The lung tissues of four mice in each group were used to prepare tissue homogenate. Then total protein in the tissue was extracted by using RIPA lysis buffer containing PMSF (R0010, Solarbio). Protein concentration was measured according to the instructions of BCA kit (Thermo, U.S.A.) and adjusted. The protein was mixed with 5× loading buffer and denatured on the boiling water bath. SDS/PAGE was used to separate the denatured protein which was then transferred to PVDF membrane. Then the membrane was sealed with 5% skim milk at room temperature for 1.5 h and incubated at 4°C overnight with primary antibodies including rabbit anti-human TLR4 (1:1000, ab13867, Abcam, U.S.A.), MyD88 (1:1000, ab135693, Abcam, U.S.A.), NF-κB p50 (1 μg/ml, ab220803, Abcam, U.K.), p-NF-κB p50 (1 μg/ml, phospho S337, ab28849, Abcam, U.K.), Bax (1:1000, ab8805, Abcam, U.S.A.), Bcl-2 (1:1000, ab32124, Abcam, U.S.A.) and GAPDH (1:5000, ab9385, Abcam, U.S.A.). After the membrane was washed three times, it was added with horseradish peroxidase-labeled goat anti-rabbit IgG antibody (1:10000, ab97051, Abcam, U.K.) and incubated for 2 h. Then the membrane was washed three times and developed. Imaging was implemented by Bio-Rad gel imager (Bio-Rad, U.S.A.). Relative protein expression = gray value of target protein band/gray value of GAPDH band.

### HE staining

In each group, the lung tissues of four mice were fixed in 10% formalin for 24 h, which were routinely processed to prepare dewaxed sections. The section was stained using Hematoxylin (H8070-5g, Solarbio, China) for 3 min and rinsed for 5 min, differentiated using 0.5% hydrochloric acid alcohol for 10 s, rinsed with anti-blue liquid for 10 min, and stained with Eosin (G1120, Solarbio, China) for 5 min. Finally, the section was routinely dehydrated, transparentized and sealed with natural gum. An optical microscope (XP-330, Shanghai Bingyu Optical Instrument Co., Ltd., China) was employed to observe the changes of histological structure in each section.

### ELISA

The whole blood samples of four mice in each group were centrifuged at 3500 rpm and 4°C for 15 min to obtain serum samples. Content of serum IL-2 (ab10752, Abcam, U.S.A.), IL-10 (ab108870, Abcam, U.S.A.) and TNF-α (ab6671, Abcam, U.S.A.) were measured according to the instructions of the kits.

### TUNEL

The paraffin section was dewaxed, hydrated, immersed in 3% H_2_O_2_ for 12 min and incubated at room temperature with proteinase K (20 μg/ml, dissolved in Tris/HCl) for 30 min. The section was rinsed three times with PBS and TUNEL reaction mixture was dropwise added on the section and incubated in a wet box at 37°C for 1 h. After, rinsing three times with PBS, and the section was observed under fluorescence microscope (ECLIPSE Ti, Nikon, Japan). Brown-stained cells represented TUNEL-positive cells. The percentage of TUNEL positive cells in total cells was counted in three randomly selected visual fields, namely apoptosis index (AI).

### Statistical analysis

The data were analyzed by SPSS 21.0 software (SPSS Inc, Chicago, IL, U.S.A.). All measurement data were shown as mean ± standard deviation. Comparison among groups was performed by one-way analysis of variance combined with post hoc Bonferroni pairwise comparison. There was a significant difference at *P*<0.05.

## Results

### Pathologic changes of the lung tissue

**Figure 1:**
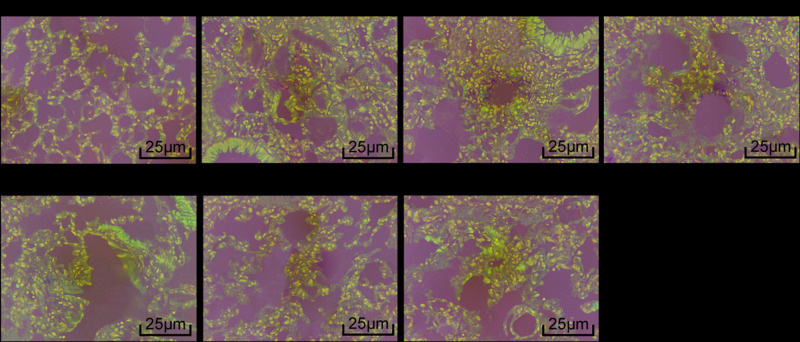
Pathologic changes of the lung tissue (400×)

Pathologic changes of the lung tissue were detected by HE staining (Figure 1). Mice in normal group had clear lung tissue and no inflammatory cell infiltration in the bronchi. Mice in model, NC and miR-143-3p inhibitor + TAK-242 groups had lymphocytes, eosinophilic granulocytes and other inflammatory cells aggregation in the tracheas. Mice in miR-143-3p mimic and TAK-242 groups had fewer inflammatory cells in the lumen of the trachea as compared with model group, while mice in miR-143-3p inhibitor group had more inflammatory cells.

### Content of IL-2, IL-10 and TNF-α in the serum

**Figure 2:**
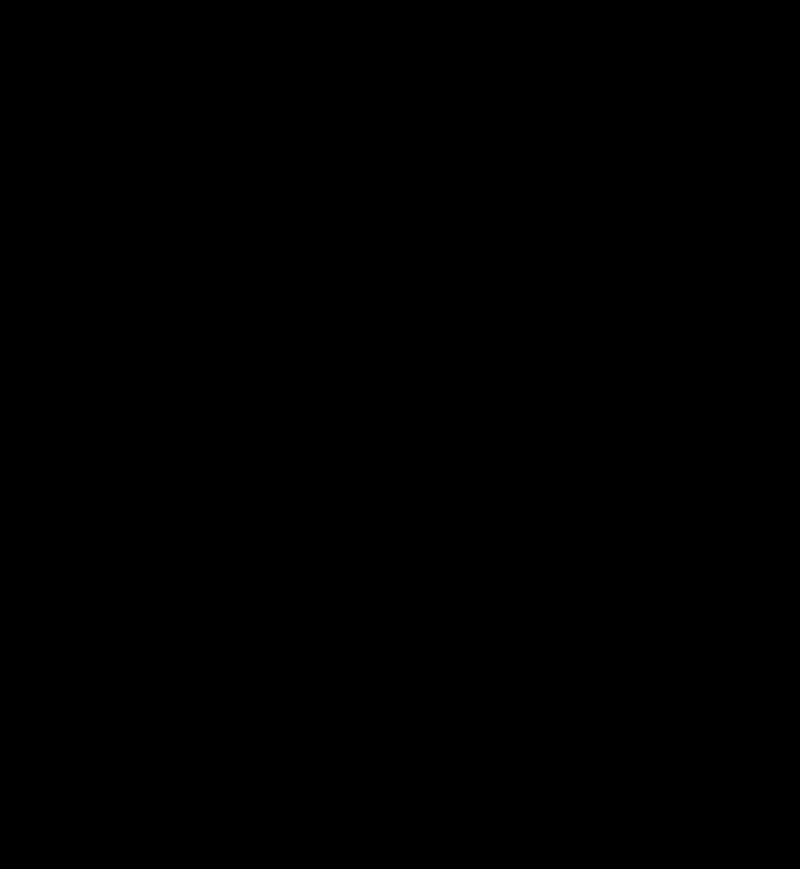
Content of IL-2, IL-10 and TNF-α in the serum Compared with normal group, *P<0.05; compared with model group, ^#^*P*<0.05; compared with NC group, ^%^*P*<0.05; compared with miR-143-3p mimic group, ^&^*P*<0.05; compared with miR-143-3p inhibitor group, ^$^*P*<0.05; compared with TAK-242 group, ^@^*P*<0.05.

Content of IL-2, IL-10 and TNF-α in the serum were detected by ELISA (Figure 2). Compared with normal group, model mice had significantly higher serum IL-2 and TNF-α content and lower IL-10 content (all *P*<0.05). Compared with mice without treatment, mice with miR-143-3p mimic and TAK-242 treatment had decreased IL-2 and TNF-α content and increased IL-10 content, while miR-143-3p inhibitor treatment induced opposite changes (all *P*<0.05). TAK-242 could reversed the changes caused by miR-143-3p inhibitor (all *P*<0.05).

### Epithelial cell apoptosis in the lung tissue

**Figure 3:**
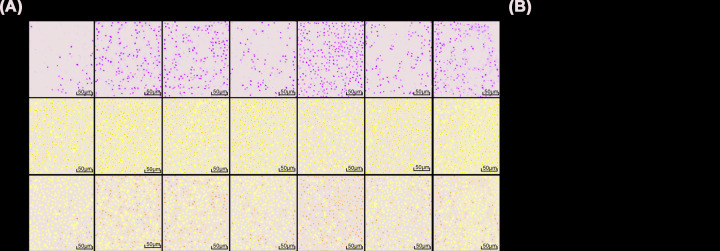
Apoptosis in the lung tissue (**A**) Apoptosis in the lung tissue detected by TUNEL staining (200×). (**B**) Number of TUNEL-positive cells in the lung tissue. Compared with normal group, *P<0.05; compared with model group, ^#^*P*<0.05; compared with NC group, ^%^*P*<0.05; compared with miR-143-3p mimic group, ^&^*P*<0.05; compared with miR-143-3p inhibitor group, ^$^*P*<0.05; compared with TAK-242 group, ^@^*P*<0.05.

Epithelial cell apoptosis in the lung tissue were detected by TUNEL staining (Figure 3). Compared with normal group, model mice had significantly more TUNEL-positive cells in the rest groups (all *P*<0.05). Compared with mice without treatment, model mice with miR-143-3p mimic and TAK-242 treatment had less TUNEL-positive cells, while miR-143-3p inhibitor treatment induced opposite changes (all *P*<0.05). TAK-242 could reversed the changes caused by miR-143-3p inhibitor (all *P*<0.05).

### Bax and Bcl-2 mRNA and protein expressions in the lung tissue

**Figure 4:**
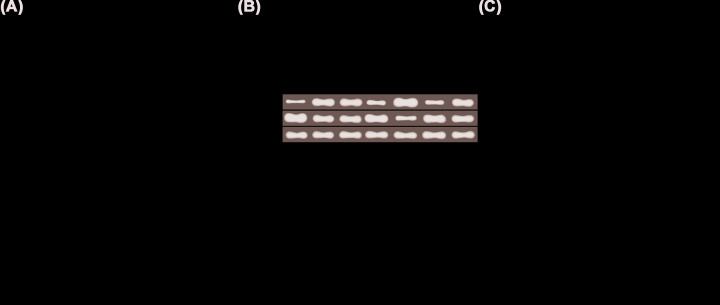
Bax and Bcl-2 mRNA and protein expressions in the lung tissue (**A**) Bax and Bcl-2 mRNA expressions, (**B**) Bax and Bcl-2 protein bands, (**C**) Bax and Bcl-2 protein expressions. Compared with normal group, *P<0.05; compared with model group, ^#^*P*<0.05; compared with NC group, ^%^*P*<0.05; compared with miR-143-3p mimic group, ^&^*P*<0.05; compared with miR-143-3p inhibitor group, ^$^*P*<0.05; compared with TAK-242 group, ^@^*P*<0.05.

mRNA and protein expressions of apoptosis-related factors Bax and Bcl-2 were measured by qRT-PCR and Western blot in order to investigate how miR-143-3p mediated TLR4/MyD88/NF-κB signaling pathway to work on the apoptosis of alveolar epithelial cells of mice with mycoplasmal pneumonia (Figure 4). Compared with normal group, model mice decreased mRNA and protein expressions of Bcl-2 and increased mRNA and protein expressions of Bax in the rest groups (all *P*<0.05).

Compared with mice without treatment, mice with miR-143-3p mimic and TAK-242 treatment had significantly higher mRNA and protein expressions of Bcl-2 and lower mRNA and protein expressions of Bax, while miR-143-3p inhibitor treatment induced opposite changes (all *P*<0.05). TAK-242 could reverse the changes caused by miR-143-3p inhibitor (all *P*<0.05).

### miR-143-3p inhibited TLR4/MyD88/NF-κB signaling pathway expression

**Figure 5:**
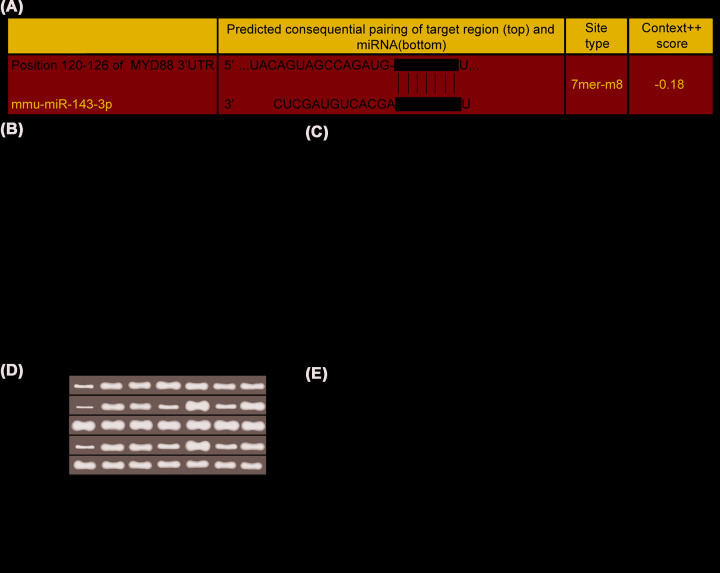
miR-143-3p inhibited the expression of TLR4/MyD88/NF-κB signaling pathway (**A**) Sequence of 3′-UTR region in which miR-143-3p bound with MYD88. (**B**) Dual-luciferase reporter system assay verified the target relationship between miR-143-3p and MYD88. Compared with NC mimic, ^Φ^*P*<0.05. (**C**) Expressions of miR-143-3p as well as TLR4, MyD88 and NF-κB p50 mRNA, (**D**) protein bands of TLR4, MyD88, NF-κB p50 and p-NF-κB p50, (**E**) protein expressions of TLR4, MyD88, NF-κB p50 and p-NF-κB p50. Compared with normal group, *P<0.05; compared with model group, ^#^*P*<0.05; compared with NC group, ^%^*P*<0.05; compared with miR-143-3p mimic group, ^&^*P*<0.05; compared with miR-143-3p inhibitor group, ^$^*P*<0.05; compared with TAK-242 group, ^@^*P*<0.05.

There was a targeted binding site between miR-143-3p and MyD88 through the prediction on the bioinformatics website (www.targetscan.org), and the target relationship between them was further verified by dual-luciferase reporter assay (Figure 5A,B). The results showed that there were no significantly differences in relative dual-luciferase activity after Wt-MYD88 and Mut-MYD88 were co-transfected with NC mimic, respectively (*P*>0.05). The relative dual-luciferase activity in wt-MYD88 group was significantly decreased as compared with Mut-MYD88 group after co-transfection with miR-143-3p mimic (*P*<0.05).

The expressions of related factors in the lung tissue were measured to further define the regulatory relations between miR-143-3p and TLR4/MyD88/NF-κB signaling pathway (Figure 5C–E). Compared with normal group, model mice had significantly decreased miR-143-3p expression, increased mRNA and protein expressions of TLR4, MyD88 and p-NF-κB p50, and unaltered NF-κB p50 protein expression (all *P*<0.05).

Compared with model group, MyD88 and NF-κB expressions significantly decreased in miR-143-3p mimic and TAK-242 groups, and increased in miR-143-3p inhibitor group; TLR4 mRNA and protein expressions significantly decreased in TAK-242 and miR-143-3p inhibitor + TAK-242 groups; miR-143-3p increased in miR-143-3p mimic group and reduced in miR-143-3p inhibitor group and miR-143-3p inhibitor + TAK-242 group (all *P*<0.05).

## Discussion

MP can cause acute bronchitis, community-acquired pneumonia and acute asthma and children and young adults are susceptible population [20]. There is increasing evidence that MP-induced symptoms of pneumonia are associated with pro-inflammatory cytokines’ expressions and the induction of pulmonary fibrosis [21]. MP infection can lead to the increase in pro-inflammatory cytokines, TNF-α and chemokine, such as IL-6 and promotes various leukocytes (mainly neutrophils) aggregation at the infection site, eventually resulting in lung injuries and pulmonary fibrosis [22]. Lipoproteins and lipopeptides from *Mycoplasma* species are important in triggering innate immune responses to the organism, and TLR2 is a molecule that binds to lipoproteins and lipopeptides derived from these *Mycoplasma* species. The binding of receptor and ligand leads to the activation of TLR2 signal transduction pathway and the production of pro-inflammatory cytokines [23]. MP is mainly treated with antibiotics (like doxycycline, fluoroquinolones or azithromycin), glucocorticoids and intravenous immunoglobulins, which often causes adverse reactions [23]. Therefore, it is necessary to explore new targets.

miRNA has been reported to target proto-oncogenes, such as Ras, Bcl-2 or c-Myc [24]. Bcl-2 and Bax belong to the anti-apoptosis Bcl-2 family, which can inhibit caspase-3 activation and play key roles in the regulation of apoptosis [25]. It is reported that miR-143-3p mediates cytokine-induced killer cell proliferation [26]. miR-143-3p significantly inhibited the production of IL-1β, IL-6, IL-8, MMP-1 and MMP-13 [27]. Changes in miR-143-3p expression have been observed in multiple human diseases, such as arthritis [28]. TLRs are important in the innate immune system, of which TLR4 initiates a series of signal transductions [29]. LPS-induced inflammatory process is mediated by TLR4 [30]. TLR4 recognizes LPS and initiates a series of cascade reactions, including the activation of IRAK by MyD88; subsequently, IRAK4/IRAK1/MyD88 signaling complexes lead to the activation of IKKα, initiating the phosphorylation degradation of IκB-α and NF-κB [31]. NF-κB, an important regulatory factor of inflammatory diseases, promotes the transcription of IL-2, IL-10 and TNF-α [32,33]. In a word, TLR4/MyD88/NF-κB signaling pathway is activated in mice with lung injuries [34].

In the present study, miR-143-3p expression was down-regulated, and the expressions of TLR4, MyD88 and NF-κB were up-regulated in MP mice with acute lung injury. Administration of TLR4 inhibitor could inhibit MyD88 and NF-κB expressions, lower IL-2 and TNF-α content, increase IL-10 content, reduce alveolar epithelial cell apoptosis, decrease Bax expression and increase Bcl-2 expression in MP mice, which was consistent with previous reports. These results confirmed the treatment effect of target inhibition of TLR4/MyD88/NF-κB signaling pathway on lung injury in mice.

Via bioinformatics website, we found that miR-143-3p may be the upstream molecule of TLR4/MyD88/NF-κB signaling pathway and the dual-luciferase reporter system assay confirmed that miR-143-3p regulated MyD88 negatively. In MP model mice, miR-143-3p up-regulation inhibited the release of inflammatory factors and alveolar epithelial cell apoptosis, while miR-143-3p inhibitor had opposite effects. Moreover, the deterioration caused by miR-143-3p inhibitor can be reversed by TAK-242, which is the inhibitor of TLR4/MyD88/NF-κB signaling pathway. Therefore, we determined that miR-143-3p could negatively regulate MyD88/NF-κB signaling pathway expression in a targeted way, thereby inhibiting the abnormal inflammation and reducing the apoptosis of alveolar epithelial cells induced by mycoplasmal pneumonia in mice.

Through the above studies, we confirmed miR-143-3p could improve mycoplasmal pneumonia by inhibiting inflammation response and cell apoptosis of alveolar epithelial cells in mice, and the mechanism may be that miR-143-3p can negatively regulate MyD88 expression in a targeted way, thus to negatively regulate TLR4/MyD88/NF-κB signaling pathway. It is noteworthy that in the present study, all indexes in miR-143-3p inhibitor + TAK-242 group recovered significantly, but not to the levels of TAK-242 group. Therefore, we speculate that another target site of miR-143-3p regulates mycoplasmal pneumonia, which has not been confirmed yet.

## Abbreviations

Bax: Bcl2-associated X protein
Bcl-2: B-cell lymphoma 2
GAPDH: glyceraldehyde-3-phosphate dehydrogenase
HE: hematoxylin-eosin
IL: interleukin
IKK: IκB kinase
IRAK: interleukin-1 receptor-associated kinase
LPS: lipopolysaccharide
miRNA: microRNA
MMPs: matrix metalloproteinase
MP: *Mycoplasma pneumoniae*
MyD88: myeloid differentiation factor 88
NC: negative control
PPAR: peroxisome proliferator-activated receptor
PGL3: pGL3 Luciferase Reporter Vector
qRT-PCR: quantitative reverse transcription-polymerase chain reaction
RIPA: radioimmunoprecipitation assay
TUNEL: terminal deoxynucleotidyl transferase-mediated dUTP-biotin nick end labeling
TLR: Toll-like receptor
TNF: tumor necrosis factor

## References

[1] Lee H., Yun K.W., Lee H.J. and Choi E.H. (2018) Antimicrobial therapy of macrolide-resistant *Mycoplasma pneumoniae* pneumonia in children. Exp. Rev. Anti Infect. Ther. 16, 23–34 10.1080/14787210.2018.1414599 29212389

[2] Meng Y.L., Wang W.M., Lv D.D., An Q.X., Lu W.H., Wang X. et al. (2017) The effect of Platycodin D on the expression of cytoadherence proteins P1 and P30 in Mycoplasma pneumoniae models. Environ. Toxicol. Pharmacol. 49, 188–193 10.1016/j.etap.2017.01.001 28073091

[3] Liu F., Zhang X., Zhang B., Mao W., Liu T., Sun M. et al. (2018) TREM1: A positive regulator for inflammatory response via NF-kappaB pathway in A549 cells infected with Mycoplasma pneumoniae. Biomed. Pharmacother. 107, 1466–1472 30257363 10.1016/j.biopha.2018.07.176

[4] Chmura K., Bai X., Nakamura M., Kandasamy P., McGibney M., Kuronuma K. et al. (2008) Induction of IL-8 by Mycoplasma pneumoniae membrane in BEAS-2B cells. Am. J. Physiol. Lung Cell. Mol. Physiol. 295, L220–L230 10.1152/ajplung.90204.2008 18487355 PMC2494795

[5] Ledford J.G., Mukherjee S., Kislan M.M., Nugent J.L., Hollingsworth J.W. and Wright J.R. (2012) Surfactant protein-A suppresses eosinophil-mediated killing of Mycoplasma pneumoniae in allergic lungs. PLoS ONE 7, e32436 10.1371/journal.pone.0032436 22384248 PMC3285686

[6] Segovia J.A., Chang T.H., Winter V.T., Coalson J.J., Cagle M.P., Pandranki L. et al. (2018) NLRP3 is a critical regulator of inflammation and innate immune cell response during Mycoplasma pneumoniae infection. Infect. Immun. 86, e00548–17 29061706 10.1128/IAI.00548-17 PMC5736809

[7] Essandoh K., Li Y., Huo J. and Fan G.C. (2016) MiRNA-mediated macrophage polarization and its potential role in the regulation of inflammatory response. Shock 46, 122–131 10.1097/SHK.0000000000000604 26954942 PMC4949115

[8] Shi S., Liu X. and Li H. (2017) Downregulation of caspase3 alleviates Mycoplasma pneumoniaeinduced apoptosis in alveolar epithelial cells. Mol. Med. Rep. 16, 9601–9606 10.3892/mmr.2017.7782 29039549

[9] He Z., Yi J., Liu X., Chen J., Han S., Jin L. et al. (2016) MiR-143-3p functions as a tumor suppressor by regulating cell proliferation, invasion and epithelial-mesenchymal transition by targeting QKI-5 in esophageal squamous cell carcinoma. Mol. Cancer 15, 51 10.1186/s12943-016-0533-3 27358073 PMC4928305

[10] Yu B., Zhao Y., Zhang H., Xie D., Nie W. and Shi K. (2018) Inhibition of microRNA-143-3p attenuates myocardial hypertrophy by inhibiting inflammatory response. Cell Biol. Int. 42, 1584–1593 10.1002/cbin.11053 30203887

[11] Yang Z., Wang J., Pan Z. and Zhang Y. (2018) miR-143-3p regulates cell proliferation and apoptosis by targeting IGF1R and IGFBP5 and regulating the Ras/p38 MAPK signaling pathway in rheumatoid arthritis. Exp. Ther. Med. 15, 3781–3790 29581736 10.3892/etm.2018.5907 PMC5863597

[12] Ding X., Jin S., Tong Y., Jiang X., Chen Z., Mei S. et al. (2017) TLR4 signaling induces TLR3 up-regulation in alveolar macrophages during acute lung injury. Sci. Rep. 7, 34278 10.1038/srep34278 28198368 PMC5309825

[13] Kim S.H., Bang J., Son C.N., Baek W.K. and Kim J.M. (2018) Grape seed proanthocyanidin extract ameliorates murine autoimmune arthritis through regulation of TLR4/MyD88/NF-kappaB signaling pathway. Korean J. Intern. Med. 33, 612–621 10.3904/kjim.2016.053 27271273 PMC5943648

[14] Kong F., Ye B., Cao J., Cai X., Lin L., Huang S. et al. (2016) Curcumin represses NLRP3 inflammasome activation via TLR4/MyD88/NF-kappaB and P2X7R signaling in PMA-induced macrophages. Front. Pharmacol. 7, 369 10.3389/fphar.2016.00369 27777559 PMC5056188

[15] Jiang Q., Yi M., Guo Q., Wang C., Wang H., Meng S. et al. (2015) Protective effects of polydatin on lipopolysaccharide-induced acute lung injury through TLR4-MyD88-NF-kappaB pathway. Int. Immunopharmacol. 29, 370–376 10.1016/j.intimp.2015.10.027 26507165

[16] Zhu H.T., Bian C., Yuan J.C., Chu W.H., Xiang X., Chen F. et al. (2014) Curcumin attenuates acute inflammatory injury by inhibiting the TLR4/MyD88/NF-kappaB signaling pathway in experimental traumatic brain injury. J. Neuroinflammation 11, 59 10.1186/1742-2094-11-59 24669820 PMC3986937

[17] Zhang R., Ai X., Duan Y., Xue M., He W., Wang C. et al. (2017) Kaempferol ameliorates H9N2 swine influenza virus-induced acute lung injury by inactivation of TLR4/MyD88-mediated NF-kappaB and MAPK signaling pathways. Biomed. Pharmacother. 89, 660–672 28262619 10.1016/j.biopha.2017.02.081

[18] Meng L., Li L., Lu S., Li K., Su Z., Wang Y. et al. (2018) The protective effect of dexmedetomidine on LPS-induced acute lung injury through the HMGB1-mediated TLR4/NF-kappaB and PI3K/Akt/mTOR pathways. Mol. Immunol. 94, 7–17 10.1016/j.molimm.2017.12.008 29241031

[19] Yu Y., Chen Y., Wang Y., Li Y., Zhang L. and Xin J. (2018) TLR2/MyD88/NF-kappaB signaling pathway regulates IL-1beta production in DF-1cells exposed to Mycoplasma gallisepticum LAMPs. Microb. Pathog. 117, 225–231 10.1016/j.micpath.2018.02.037 29471139

[20] Maselli D.J., Medina J.L., Brooks E.G., Coalson J.J., Kannan T.R., Winter V.T. et al. (2018) The immunopathologic effects of Mycoplasma pneumoniae and community-acquired respiratory distress syndrome toxin. A primate model. Am. J. Respir. Cell Mol. Biol. 58, 253–260 10.1165/rcmb.2017-0006OC 28915064 PMC5805996

[21] Kurai D., Nakagaki K., Wada H., Saraya T., Kamiya S., Fujioka Y. et al. (2013) Mycoplasma pneumoniae extract induces an IL-17-associated inflammatory reaction in murine lung: implication for mycoplasmal pneumonia. Inflammation 36, 285–293 10.1007/s10753-012-9545-3 23001692

[22] Lin Y., Tan D., Kan Q., Xiao Z. and Jiang Z. (2018) The protective effect of naringenin on airway remodeling after Mycoplasma pneumoniae infection by inhibiting autophagy-mediated lung inflammation and fibrosis. Mediators Inflamm. 2018, 8753894 10.1155/2018/8753894 29849498 PMC5904783

[23] Yan Y., Wei Y., Jiang W. and Hao C. (2016) The clinical characteristics of corticosteroid-resistant refractory Mycoplasma Pneumoniae pneumonia in children. Sci. Rep. 6, 39929 10.1038/srep39929 28008989 PMC5180238

[24] Zhou L.L., Zhu Y.M., Qian F.Y., Yuan C.C., Yuan D.P. and Zhou X.P. (2018) MicroRNA1433p contributes to the regulation of pain responses in collageninduced arthritis. Mol. Med. Rep. 18, 3219–3228 30066874 10.3892/mmr.2018.9322 PMC6102648

[25] Mu S., Kang B., Zeng W., Sun Y. and Yang F. (2016) MicroRNA-143-3p inhibits hyperplastic scar formation by targeting connective tissue growth factor CTGF/CCN2 via the Akt/mTOR pathway. Mol. Cell. Biochem. 416, 99–108 10.1007/s11010-016-2699-9 27075467

[26] Tiedt S., Prestel M., Malik R., Schieferdecker N., Duering M., Kautzky V. et al. (2017) RNA-Seq identifies circulating miR-125a-5p, miR-125b-5p, and miR-143-3p as potential biomarkers for acute ischemic stroke. Circ. Res. 121, 970–980 10.1161/CIRCRESAHA.117.311572 28724745

[27] Li D., Hu J., Song H., Xu H., Wu C., Zhao B. et al. (2017) miR-143-3p targeting LIM domain kinase 1 suppresses the progression of triple-negative breast cancer cells. Am. J. Transl. Res. 9, 2276–2285 28559978 PMC5446510

[28] Miyashita T., Ahmed A.K., Nakanuma S., Okamoto K., Sakai S., Kinoshita J. et al. (2016) A three-phase approach for the early identification of acute lung injury induced by severe sepsis. In Vivo 30, 341–349 27381595

[29] Liu W., Liu K., Zhang S., Shan L. and Tang J. (2018) Tetramethylpyrazine showed therapeutic effects on sepsis-induced acute lung injury in rats by inhibiting endoplasmic reticulum stress protein kinase RNA-like endoplasmic reticulum kinase (PERK) signaling-induced apoptosis of pulmonary microvascular endothelial cells. Med. Sci. Monit. 24, 1225–1231 29488473 10.12659/MSM.908616 PMC5841188

[30] Chen Z., Ding X., Jin S., Pitt B., Zhang L., Billiar T. et al. (2016) WISP1-alphavbeta3 integrin signaling positively regulates TLR-triggered inflammation response in sepsis induced lung injury. Sci. Rep. 6, 28841 10.1038/srep28841 27349568 PMC4923866

[31] Park I., Kim M., Choe K., Song E., Seo H., Hwang Y. et al. (2019) Neutrophils disturb pulmonary microcirculation in sepsis-induced acute lung injury. Eur. Respir. J. 53, 1800786 10.1183/13993003.00786-2018 30635296 PMC6437604

[32] Xue M., Sun Z., Shao M., Yin J., Deng Z., Zhang J. et al. (2015) Diagnostic and prognostic utility of tissue factor for severe sepsis and sepsis-induced acute lung injury. J. Transl. Med. 13, 172 10.1186/s12967-015-0518-9 26025445 PMC4459056

[33] Sun Y., Sun L., Liu S., Song J., Cheng J. and Liu J. (2015) Effect of emodin on Aquaporin 5 expression in rats with sepsis-induced acute lung injury. J. Tradit. Chin. Med. 35, 679–684 26742314 10.1016/s0254-6272(15)30159-x

[34] Huang C., Pan L., Lin F., Dai H. and Fu R. (2017) Monoclonal antibody against Toll-like receptor 4 attenuates ventilator-induced lung injury in rats by inhibiting MyD88- and NF-kappaB-dependent signaling. Int. J. Mol. Med. 39, 693–700 10.3892/ijmm.2017.2873 28204830

