# Expression of Concern: miR-143-3p impacts on pulmonary inflammatory factors and cell apoptosis in mice with mycoplasmal pneumonia by regulating TLR4/MyD88/NF-κB pathway

**DOI:** 10.1042/BSR-20193419_EOC

**Published:** 2021-03-31

**Keywords:** apoptosis, inflammation, miR-143-3p, mycoplasmal pneumonia, TLR4/MyD88/NF-κB signaling pathway

The Editorial Office has been made aware of potential issues surrounding the scientific validity of this paper, hence has issued an expression of concern to notify readers whilst the Editorial Office investigates.

